# A Mysterious Pacemaker Suture: An Uncommon Foreign Body Reaction

**Published:** 2011-02-08

**Authors:** Naveen Garg, Nagaraja Moorthy

**Affiliations:** Department of Cardiology, Sanjay Gandhi Postgraduate Institute of Medical Sciences, Lucknow, India

**Keywords:** permanent pacemaker, surgical suture, foreign body reaction

## Abstract

Surgical suture material is usually inert and nontoxic and causes minimal inflammation of tissue. However, foreign body reactions to various suture types can lead to granuloma, abscess, or even sinus formation. We report an elderly female who was incidentally detected to have a mass protruding from the incision site which was confirmed histopathologically a chronic granulomatous reaction to non absorbable suture. The foreign body granulomatous reaction to suture material in the setting of pacemaker implantation has not been described in the literature. We also discuss the existing literature on this underrecognised entity.

## Introduction

Surgical suture material is usually inert and nontoxic and causes minimal inflammation of tissue. However, there are case reports of foreign body reactions to various suture types leading to granuloma, abscess, or even sinus formation. Little is known of the body's response to various suture materials. Biologic reactivity to suture materials can have an effect on wound healing and patient outcomes.

## Case report

A 65 year old female, presented to cardiology out patient department with history of recent onset recurrent presyncope. She gave history of syncope around 10 years back and was diagnosed to have intermittent complete heart block for which she underwent permanent pacemaker (single chamber-VVI mode) implantation through right subclavian vein approach. She was nondiabetic and normotensive and never had history suggestive of coronary artery disease. Her clinical examination revealed bradycardia and electrocardiography revealed complete heart block with ventricular rate of 32/min. Pacemaker interrogation confirmed pacemaker battery end of life status.

**Figure 1:**
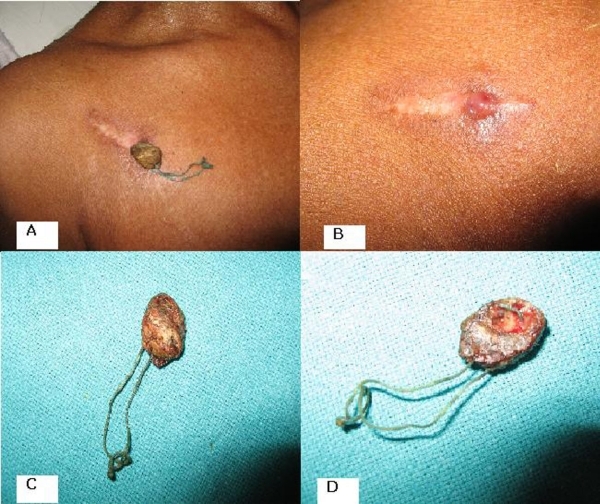
Panel A: Growth from the incision site with extrusion of the suture material; Panel B: Post excision image showing the site of attachment of mass; Panel C: Excised mass in ventral view; Panel D: Excised mass in dorsal view

The pacemaker pocket site examination revealed an unusual fleshy mass (Figure 1) measuring 2x1x1cm which was protruding through the medial end of incision. The nonabsorbable suture material was traversing through the mass and was entirely exposed to the environment. There were no signs of inflammation or tenderness. Routine blood investigations were normal. On enquiring she revealed that, she noticed a peanut size mass which was gradually growing in size over last ten years along with the extrusion of the suture material. There was no pain or bloody or purulent discharge from the mass hence she did not seek any medical attention. She never attended pacemaker clinic for pacemaker interrogation since the time of implantation.

**Figure 2:**
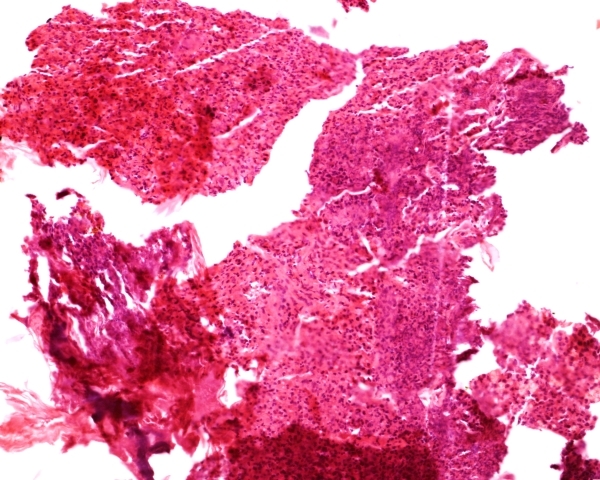
Microscopic examination (H&E stain) showing extensive lymphocytic infiltration with occasional giant cells

In view of recurrent syncope and complete heart block immediate temporary pacemaker implantation was done through right femoral route and planned for pulse generator replacement. The mass was excised in toto along with the suture material. The mass was firm in consistency and was easily separable from the skin. She underwent successful pulse generator replacement through the same site. Later the histopathological examination of the tissue confirmed chronic granulamatous inflammation with extensive lymphocytic infiltration with occasional giant cells (Figure 2).There was no evidence of malignancy. The culture and staining of the tissue for bacteriae and fungus were negative. During six months of follow-up she is asymptomatic and there was no recurrence of the mass.

## Discussion

The rate of implantation of pacemakers and implantable cardioverter-defibrillators (ICDs) is ever-increasing. This increase parallels the potential for more widespread indications pending the results of ongoing trials of pacing in heart failure and sudden death prevention. The relative ease of device implantation utilizing a relatively simple, expeditious, percutaneous approach, without the requirement for general anesthesia or long recuperation times, has fueled enthusiasm for implantation.

Complications related to pacemaker implantation may be surgical/hardware, programming/ software, or normal device function related. Pacemaker specific complications include failure to pace, failure to sense, pulse generator failure, pacemaker syndrome and pacemaker mediated tachycardia. The pacemaker pocket related complications include haematoma, wound pain, skin erosion and pacemaker infection.We describe an unusual delayed pacemaker pocket related complication presenting as foreigh body granuloma. Chronic inflammatory reaction to suture material was considered as the possible cause for this uncommon phenomenon.

Suture materials are indispensable implants of all types of surgeries. All surgical suture materials cause some degree of inflammatory reaction. Suture material that elicits a severe and prolonged inflammatory reaction can affect negatively the healing process and render a wound more susceptible to infection. Foreign-body excretion is a bioresponse of the human body. A foreign body granuloma is a reaction to exogenous (foreign) or endogenous materials that are too large to be ingested by macrophages. These localized lesions may occur at any age and clinically present as papules, plaques or nodules.

Various foreign bodies introduced into the human organism during surgery or trauma as well as exposure to some chemical substances may cause a granulomatous reaction [1]. Although rare, foreign body granulomas may cause diagnostic controversy when they present with neoplasia-like imaging findings. Tissue reactivity, infection and wound dehiscence rate may be influenced by patient-related factors, e.g., diabetes, overweight, malignoma, compliance. None of these factors were noted in our patients. Postoperative inflammatory reaction [2] and acute hypersensitivity [3] to suture material are seen within 48 hours after the operation.Continuous subcuticular suture has been favoured by some authors when compared to percutaneous skin sutures [4].

Nonabsorbable silk sutures have been a frequently used foreign material in surgery. In general, they are reliable and safe with minimal bio-incompatibility. Generally, it causes a granulomatous reaction with variable rates and extents of absorption. This response is usually not clinically apparent and does not interfere with successful wound healing. Delayed reactions are very rare [5]. In our patient the incision was closed with silk and she developed delayed hypersensitivity reaction in the form of nodular granulomatous lesion along with extrusion of the suture through incision site which is very rare. The extrusion of suture may increase the risk of infection but surprisingly our patient did not develop pacemaker pocket infection inspite of having the suture being exposed to exterior for years. With the increasing use of the absorbable synthetic polymers, the foreign-body reactions seen with the use of silk will become rarer. In such cases, successful treatment may occur only after removal of the silk sutures. The foreign body reaction to suture materials is an underrecognised complication which may be difficult to differentiate from pacemaker pocket infection, pocket erosion, wound dehiscence etc.

## Conclusion

Foreign-body reaction to sutures should be included in the differential diagnosis of patients who present with wound breakdown or apparent wound infection. Such reactions can present as nodular mass with extrusion of suture material. Foreign body reaction to suture material is applicable even in the setting of permanent pacemaker implantation and should be considered in the early pacemaker pocket infection, wound dehiscence or delayed wound healing because removal of the suture material only can promote early healing.
